# Nanocrystals of Poorly Soluble Drugs: Drug Bioavailability and Physicochemical Stability

**DOI:** 10.3390/pharmaceutics10030134

**Published:** 2018-08-21

**Authors:** Maria Rosa Gigliobianco, Cristina Casadidio, Roberta Censi, Piera Di Martino

**Affiliations:** School of Pharmacy, University of Camerino, via S. Agostino, 1, 62032 Camerino, Italy; maria.gigliobianco@unicam.it (M.R.G.); cristina.casadidio@unicam.it (C.C.); piera.dimartino@unicam.it (P.D.M.)

**Keywords:** nanocrystals, poorly soluble drug, nanotechnology, stability, drug delivery, drug targeting

## Abstract

Many approaches have been developed over time to overcome the bioavailability limitations of poorly soluble drugs. With the advances in nanotechnology in recent decades, science and industry have been approaching this issue through the formulation of drugs as nanocrystals, which consist of “pure drugs and a minimum of surface active agents required for stabilization”. They are defined as “carrier-free submicron colloidal drug delivery systems with a mean particle size in the nanometer range, typically between 10–800 nm”. The primary importance of these nanoparticles was the reduction of particle size to nanoscale dimensions, with an increase in the particle surface area in contact with the dissolution medium, and thus in bioavailability. This approach has been proven successful, as demonstrated by the number of such drug products on the market. Nonetheless, despite the definition that indicates nanocrystals as a “carrier-free” system, surface active agents are necessary to prevent colloidal particles aggregation and thus improve stability. In addition, in more recent years, nanocrystal properties and technologies have attracted the interest of researchers as a means to obtain colloidal particles with modified biological properties, and thus their interest is now also addressed to modify the drug delivery and targeting. The present work provides an overview of the achievements in improving the bioavailability of poorly soluble drugs according to their administration route, describes the methods developed to overcome physicochemical and stability-related problems, and in particular reviews different stabilizers and surface agents that are able to modify the drug delivery and targeting.

## 1. Introduction

It is estimated that 90% of new drugs in the development pipeline can be classified as poorly soluble [1]. Given the great number of poorly soluble drugs, innovative and appropriate formulations as well as technological solutions are needed to sufficiently increase drug bioavailability, accordingly to the administration route [2].

To date, the classical approach for increasing the dissolution rate of poorly soluble drugs is to reduce particle size, in particular through micronization [3]. However, it seems that further improvement in the drug dissolution rate and thus in bioavailability demands a shifting from micronization to nanonization. This requires different and innovative technological approaches, as well as innovative solutions to overcome all of the physicochemical and stability problems associated with nanostructures. 

Thanks to the advanced process technologies and analytical methods developed in the last decades, a considerable number of pharmaceutical nanocrystal products are now on the market and several are under development [4]. Nanocrystals consisting of pure drugs and a minimum of surface active agents required for stabilization are carrier-free submicron colloidal drug delivery systems with a mean particle size in the nanometer range, typically between 10–800 nm [5]. Classically, nanocrystals were developed for their size and the minimum amount of surface active agents required for stabilization [6]. 

Nonetheless, in more recent years, nanocrystals have attracted the interest of researchers as a means of obtaining colloidal particles with modified biological properties that allow modifying the drug delivery and targeting. 

Thus, nanocrystals offer several advantages and disadvantages. Among the advantages, there are:

✓ Possibility of administering a drug through different administration routes (oral, intravenous, intramuscular, pulmonary, ocular, dermal) [7,8,9,10,11,12,13,14,15,16,17,18,19,20,21,22,23,24];
✓ Possibility of formulating a drug under different pharmaceutical dosage forms (tablets, capsules, suspensions, ointments, etc.) [17,25,26,27,28,29];
✓ Higher solubility (thermodynamic or kinetic depending on the solid physical state of the drug) than conventional particles [8,17,22,23,30,31,32];
✓ Faster dissolution rate than conventional particles [3,8,9,16,30,33,34,35];
✓ Long circulating nanocrystals [36,37,38];
✓ Potential for passive and active targeting of drugs [39,40];
✓ Reduced tissue irritation in case of subcutaneous/intramuscular administration [41];
✓ Possibility of obtaining hybrid nanocrystals for both diagnostic and therapeutic applications [24,42,43,44].
✓ Fewer are the disadvantages, such as:
✓ Physicochemical-related stability problems [4,35,45,46,47];
✓ Bulking sufficient care must be taken during handling and transport [5,48];
✓ Uniform and accurate dose cannot be achieved [49,50].

A brief summary of terminology is useful. First, the term nanocrystal has a different meaning than the term nanoparticle. “Nanocrystal” is often used indiscriminatingly to refer to the drug solid state, specifically for solid particles in the nanometric range with minimum excipients. “Nanoparticle” refers more generally to particles in the nanometric range dimension, and mainly includes polymers or lipids, such as for example polymeric nanoparticles, liposomes, solid lipid nanoparticles, etc. Thus, in the case of nanocrystals, the term “nanoparticles” is not preferred because it is ambiguous. It is true that in several cases, these “nanocrystals” exist in an amorphous physical state, but this should not justify the use of the term “crystal”. Some have avoided the ambiguity by using the term “pure solid nanoparticles” to refer to solid pure nanoparticles without any reference to the drug solid physical state [51]. When referring to formulations where nanoparticles are dispersed in a liquid, the preferred term is “nanosuspension”. In the present review, the term “nanocrystal” will be generally preferred in reference to the solid physical state, because it is generally used, accepted, and understood by the scientific community.

Lastly, the term “nanocomposite” more specifically refers to nanoparticles (nanosuspensions) subjected to a drying process to intentionally recover nanoaggregates that are able to redisperse in a liquid (reversible or irreversible aggregates) [52].

This review has three objectives:

- to overview the more recent information about the improvement in the dissolution rate and bioavailability of poorly soluble drugs, formulated as nanocrystals and administered through different administration routes;
- to review the physicochemical stability-related problems of nanocrystals and the methodological approaches to improve the physicochemical stability of formulations;
- to review the use of nanocrystal surface modifiers for different applications, from physicochemical stabilization to drug delivery and targeting.

The focus is on scientific works of the last decade, with a few exceptions. 

In this review, nanocomposites are only marginally considered, because the related topic represents a huge area of interest by itself, and thus we send to specific references [52,53,54].

## 2. Production Technologies of Nanocrystals

Nanoparticles can be produced through top-down and bottom-up technologies. Top-down approaches involve the reduction of large particles to the nanometer range, such as for example by milling, while bottom-up methods generate nanoparticles by building them from drug molecules in solution, such as by precipitation. Some approaches defined as combined technologies involve the application of two technologies in succession. It is the case for example of a combination of microprecipitation and high-pressure homogenization.

In some cases, nanoparticles generated in solution are stabilized under drying in order to ensure higher physical stability and in particular prevent aggregation, sedimentation, and creaming, allowing for the recovering of nanocomposites [52].

The drying processes applied to liquid nanosuspensions are spray-drying, freeze-drying, vacuum-drying, fluid bed coating/granulation/drying, nanoextrusion, and wet-casting drying. 

Table 1 provides a general overview of these methods.

For a detailed examination of the methods for the preparation and production of nanocrystals, the reader is encouraged to consult the following publications [32,46,55,56,57,58,59,60,61,62,63]. 

## 3. Nanocrystals (Nanosuspensions) and Bioavailability

The bioavailability of a drug depends on its ability to dissolve in biological fluids, cross membranes, and efficiently reach its pharmacological target. In the biopharmaceutical classification of drugs [94], drugs of the Class II group are characterized by poor solubility, but have a good ability to cross membranes. Thus, to improve the bioavailability of a Class II drug, it is necessary to increase drug solubility and/or the drug dissolution rate.

In particular, for nanocrystals, it is possible to consider the following scenarios:

1. A decrease in particle size leads to an increase in surface area available for the interaction with the dissolution media, and thus an increase in the particle dissolution rate, in accordance with the modified Noyes-Whitney law [7,16,95];
2. An increase in the particle curvature (particularly pronounced for colloidal particles) leads to an increase in dissolution pressure, according to the Kelvin’s equation [96];
3. Increased solubility leads to an increased concentration gradient at membranes, and thus subsequently to higher penetration or permeation through membranes [23,30,32];
4. High penetration through membranes is also favored by high adhesion to biological membranes of nanocrystals, favored by their size [97,98,99,100], but adhesion can be also improved by the coating with mucoadhesive polymer [14,28,101];
5. According to several authors, the transcellular uptake of nanocrystals through epithelial cells is another reason for the enhancement of bioavailability [9,102,103,104]. Nevertheless, Gao et al. [105] concluded that results are conflicting and confusing, and no further clarification could be highlighted;
6. Nanocrystals can be administered by intravenous injection (nanosuspensions) and are able to efficiently reach the target tissue or organ with 100% bioavailability [10];
7. Targeting can be promoted by coating nanocrystals with molecules that are able to interact with specific substrates [20,24,36,39,106,107].

As a consequence of their great potential, nanocrystals have been developed to deliver drugs under different administration routes. The following paragraphs present several examples of the most important administration routes for which a nanocrystal formulation has been developed with an improvement in drug bioavailability. 

### 3.1. Oral Drug Delivery

Oral delivery is the first choice in drug therapy, because of safety, patient compliance, ease of production, and scalability, though the principal limitations are related to drug bioavailability. Nanocrystals may improve bioavailability through an increase in solubility and particle dissolution, and through an increased gradient concentration at membranes and adhesion to the gastrointestinal wall. Classically, for Class II drugs, the rate-limiting step is the drug dissolution, and nanocrystals have been proposed to solve this limitation. 

One of the first works highlighting this concept was carried out on danazol, which is a poorly soluble drug exhibiting poor bioavailability, and was formulated as three different formulations: an aqueous nanosuspension (169 nm), a danazol-hydroxypropyl-β-cyclodextrin complex, and an aqueous microsuspension (10 μm). The area under the curve (AUC) after oral administration in beagle dogs revealed that the nanosuspension and the cyclodextrin complex had similar levels of bioavailability, while the bioavailability of the microsuspension was lower. The better performance of the aqueous nanosuspension compared to the aqueous microsuspension was explained through the former overcoming the limited dissolution rate normally observed with conventional suspensions. The authors thus proposed nanoparticles as the appropriate formulation for dissolution-rate limited absorption [26].

Following this pioneering study, many others confirmed the effectiveness of nanocrystals in improving drug bioavailability after oral administration, leading to a significant number of drug products in clinical trials or already on the market. To understand the importance of nanocrystals in the pharmaceutical field, one may note that until May 2017, the United States (US) Food and Drug Administration (FDA) has received over 80 applications for drug products containing nanocrystals, and over 60% of the submissions were for the oral route of administration [108]. 

The most recent overview of drug products in clinical trials or on the market has been reported by Arzi and Sosnik [62].

This review shows that most of the drug products are intended for an oral administration and that the pharmaceutical dosage forms are mainly tablets, capsules, or oral suspensions. In addition, one could note that the preferred technology applied is the media milling, a top-down technology that is probably better managed by the pharmaceutical industry, and that must accurately control all of the critical production factors through in-process tests and quality control end tests [108]. 

In the next section, recent examples of studies highlighting an improvement of the oral bioavailability of drugs is reported. In a lutein nanosuspension prepared by high pressure homogenization, nanocrystals exhibited a 26.3-fold increase in saturation solubility compared to that of coarse powder. The in vitro release of nanocrystals delivered in pellets and hard gelatin capsules for nutraceutical use was three to four times greater than that of coarse particles [17].

Rutin nanocrystals prepared by lyophilization and incorporated into tablets exhibited a higher particle dissolution rate than microcrystal-loaded tablets and tablets already on the market [16]. The main factor in the increased dissolution was the particle size reduction, as shown by particle size measurements achieved by photon correlation spectroscopy (PCS) and laser diffractometry (LD). 

Four nanocrystals batches (size range 80–700 nm) of coenzyme Q_10_ were prepared without any surfactant or polymer by the solvent/nonsolvent method. The dissolution rate of coenzyme Q_10_ increased as particle size decreased, and the increased bioavailability of coenzyme Q_10_ nanocrystals after oral administration was confirmed in beagle dogs where AUC_0–48_ was 4.4-fold greater than that of coarse suspensions [34].

Nanocrystals of apigenin, a bioactive flavonoid poorly soluble in water, were prepared by the supercritical antisolvent process. Nanocrystals (400–800 nm) exhibited a more rapid dissolution rate than coarse powder. After the administration of a single oral dose in rats, nanocrystals showed a significantly decreased *t*_max_, a 3.6-fold higher peak plasma concentration (*C*_max_), and a 3.4-fold higher area under the curve (AUC) than coarse particles [9].

Fenofibrate, a lipophilic drug used in hypercholesterolemia and hypertriglyceridemia, and which is practically insoluble in water, was prepared as a nanosuspension through processing in a probe sonicator and subsequent freeze-drying to transform it into a dry powder. A decrease in particle size significantly increased its saturation solubility. Pharmacokinetic studies conducted in white rabbits confirmed a 4.73-fold increase in relative bioavailability compared to the pure drug form [12]. 

Nanocrystals of bexarotene, a potent anti-tumor drug of poor solubility and bioavailability, were obtained under a method combining precipitation and microfluidization. The decreased particle size thus achieved afforded a significant increase in the dissolution rate, with improved in vivo results in rats. The higher AUC and lower *C*_max_ indicated that oral bexarotene nanocrystals significantly increased the bioavailability of this important drug and decreased its side effects. Nanocrystals administered through intravenous injection showed higher bioavailability because of the absence of first-pass effect and enterohepatic circulation [7].

Nimodipine nanocrystals of different sizes (159.0 nm, 503.0 nm, and 833.3 nm) were prepared by a combination of microprecipitation and high-pressure homogenization. The in vitro and in vivo behavior were compared to Nimotop^®^, which is a commercially available formulation of nimodipine. Even if Nimotop^®^ exhibited a higher dissolution rate than the three different nanocrystal batches, the bioavailability measured by the plasma concentration-time curves determined in beagle dogs was significantly higher for optimized nanocrystals (159.0 nm and 833.3 nm) than for Nimotop^®^ [109]. 

The in vitro/in vivo correlation for nimodipine nanocrystals and Nimotop^®^ was explained by portions of nanocrystals undergoing macropinocytosis and caveolin-mediated endocytosis by enterocytes as intact nanocrystal forms, and then bypassing the liver first-pass metabolism [9].

Similarly, no in vitro/in vivo correlation was found in the case of itraconazole solid oral nanocrystals compared to the Spranox^®^ formulation. Results showed the rapid dissolution of nanocrystals, but this behavior was not confirmed in vivo in a rat model [110]. The higher transit time of itraconazole from nanocrystals favors the rapid entrance into the small intestine where itraconazole is less stable, due to the pH at which the drug is less soluble. In Spranox^®^, the dissolution occurs from the surface of the sugar beads, which presumably exhibit longer transit times in the stomach compared to nanocrystals. The highly concentrated solution in the stomach that is actually formed with the Spranox^®^ formulation can stabilize the solution when it enters the small intestine. The stomach thus serves as a reservoir from which the highly concentrated solution can be delivered to the small intestine and be absorbed. 

It is interesting to note that several different technologies were applied to obtain nanocrystals in the previous examples, such as high pressure homogenization, lyophilization, supercritical antisolvent process, sonication followed by freeze-drying, and microprecipitation combined by high-pressure homogenization. This means that there is still a gap between the results obtained in current research and the real opportunity to apply laboratory-scale technology on an industrial scale. An important review that correlates laboratory-scale technology with their industrial feasibility was proposed by Shegokar and Müller [32].

Several evidences highlighted that the most studies on scale-up were performed on milling procedures [111,112,113].

### 3.2. Intravenous Drug Delivery

Due to their particle size, nanocrystals (nanosuspension) have the great advantage of being intravenously injectable, reaching 100% bioavailability. Nanocrystals in the range of 100–300 nm can be injected intravenously without any unwanted effect, such as the obstruction of small capillaries. Consequently, nanoparticles circulate in the bloodstream and dissolve according to their dissolution properties, and then are able to reach the target tissue. 

One of the most powerful applications of the intravenous injection of drug nanocrystal suspensions is the delivery of anticancer drugs [25,27]; even if the mechanism has not been really explained, nanocrystal formulations seem to permeate tumor tissues showing effective anticancer activity and less toxic effects than conventional formulations [43,114]. In particular, Hollis et al. [43] highlighted that the enhanced permeability and retention effect (EPR) of paclitaxel nanocrystals was comparable to that of conventional formulation, but the distribution into the tumor and organs (brain, liver, spleen, hearth) was different, probably because of a rapid accumulation of paclitaxel nanocrystals into the macrophages. Thus, even if in both formulations the drug accumulation did not exceeded 1% into the tumor, which is the dose that is considered efficacious to treat the tumor, the accumulation of paclitaxel in health organs was inferior to that of conventional formulation, explaining the lower systemic toxicity. 

In addition to these considerations, the importance of the nanocrystal size cannot be underestimated. Particles that are larger than 100 nm are mainly phagocytosed by macrophages, and thus reach the organs of the reticulum endothelial system also according to their lipophilicity, and there are only a limited number of cells able to take up these particles. However, particles smaller than 100 nm can be easily taken up by all of the cells by endocytosis [115]. The optimization of the particle size during the preparation of nanocrystals is thus of paramount importance for both biological activity and toxicity.

The formulation is also important because it affects the efficacy of the anticancer treatment. For example, Liu et al. [15] formulated paclitaxel nanocrystals in the presence of d-*R*-tocopheryl polyethylene glycol 1000 succinate as a surfactant to stabilize the nanocrystals. Those nanocrystals exhibited a variety of benefits, including a high drug-loading capacity, high stability, and sustained release. Most important, the surfactant was responsible for successfully reversing the multidrug resistance generally observed in the presence of paclitaxel formulations, in both in vitro and in vivo experiments. This effect can probably be explained by the surfactant over a certain percentage coating the paclitaxel, minimizing the interaction with those biological substrates that are responsible for multidrug resistance. Nonetheless, the paclitaxel nanocrystals exhibited improved antitumor effect compared to Taxol^®^. 

Another potent breast and lung anticancer drug, asulacrine, which is an inhibitor of topoisomerase II, was formulated as nanocrystals by high-pressure homogenization and subsequent lyophilization. The pharmacokinetic studies after intravenous administration in mice showed a good AUC in liver, lung, and kidney, and thus it was possible to predict an accumulation of the drug in some body compartments [10]. 

In a study on paclitaxel nanocrystals, Gao et al. [11] found that most of the injected dose prepared without surfactant coating was taken up by the liver (40%), while a minimal amount was present in the blood circulation and quickly eliminated. Thus, they treated the nanocrystal surface with polyethylene glycol (PEG)-based polymers and examined the impact of coating on the biodistribution, pharmacokinetics, and retention of the drug in the tumor tissue. The coating significantly enhanced the blood circulation of the drug and accumulation in tumor tissue. This approach to nanocrystals is in agreement with that generally used for other nanoparticles (liposomes and polymeric nanoparticles), where the PEG hydrophilic layer decreased the macrophage uptake, prolonging the circulation time and thus the probability that nanoparticles can reach the target tissue [29]. 

Shegokar and Singh [20] modified the surface of nanosuspension of nevirapine, an antiviral non-nucleoside reverse transcriptase inhibitor, with serum albumin, polysaccharide, and polyethylene glycol to enhance its targeting potential. The coated antiretroviral drug accumulated in various organs of rat differently than the plain drug solution did when administered intravenously. Nanosuspension showed higher mean retention time (MRT) values in brain, liver, and spleen than the plain drug. 

An improvement in drug solubility and the dissolution rate of oridonin with respect to commercial formulation was possible using a nanosuspension prepared by high-pressure homogenization in the presence of Pluronic^®^ F68, Brij^®^ 78, PVP K25, sodium dodecyl sulfate, or lecithin as stabilizers [30]. The solubility of nanocrystals was far higher than that of commercial oridonin and oridonin physical mixtures with stabilizers. This strongly impacted the drug dissolution rate. 

Fluorescent molecules have been used with nanocrystals in hybrid formulations to be applied in both therapy and diagnosis. Interestingly, Zhao et al. [24] integrated a guest fluorescent substance into the crystal lattice of a poorly soluble anticancer drug, paclitaxel, thus producing a hybrid nanosystem for the concurrent aims of tumor targeting and imaging. 

In spite of the relevant number of studies addressing solutions to treat cancer by formulating drugs as nanocrystals, no drug products are in the market, nor in clinical trial [116].

The behavior of nanocrystals after injection not being fully predictable can still be considered the main limitation to an industrial development of anticancer drug nanocrystals [117]. 

Up until now, the only drug product approved for marketing and available in an injectable dosage form is Invega^®^, which is a drug product containing the anti-depressant paliperidone palmitate, produced by high-pressure homogenization [61].

### 3.3. Pulmonary Drug Delivery

The pulmonary administration of drugs has proven highly successful not only for treating lung pathologies, but also for systemic action, because of the very large surface area available for drug adsorption, as well as the first-pass metabolism effect being avoidable. Fast onset and rapid particle deposition are other advantages. 

However, there are also drawbacks to this route, such as the limited dissolution of poorly soluble drugs, rapid clearance due to ciliary movements, less residence time and thus an absence of prolonged effect, and the unwanted deposition of particles in the pharynx and mouth [19]. While the administration of nanocrystals through this route can favorably exploit the advantages of nanocrystals and those of the pulmonary administration route [118], it has been proposed that the pulmonary delivery of nanosuspensions would be best, as it would overcome these drawbacks [19]. 

Several successful formulations have been studied.

Nanocrystals of budesonide, a poorly soluble corticosteroid anti-inflammatory drug, were prepared by wet ball milling, and subsequently loaded into hyaluronic acid microparticles by the spray-drying process. This system allowed for sustained budesonide pharmacological effects that could be explained by the mucoadhesion of the hyaluronic acid on the pulmonary mucosa, its gelation, and then the release of budesonide nanocrystals on the mucosa. The presence of mucoadhesive polymer overcame the mucociliary clearance, and consequently prolonged the retention of the active substance in the lungs [14].

Baicalein nanocrystals were prepared by a combination of antisolvent crystallization and high-pressure homogenization. Nanocrystals exhibited significantly enhanced dissolution, confirmed in vivo: pulmonary baicalein was rapidly and extensively absorbed through the pulmonary mucosa, showing pharmacokinetics parameters that were identical to those obtained after the intravenous injection of this drug [24]. 

Nanocrystalline formulations of itraconazole were prepared by wet milling and compared to amorphous itraconazole prepared by an ultra-rapid freezing process. The particle surface area was comparable, and the pulmonary delivery of amorphous itraconazole resulted in significantly higher systemic bioavailability than for the nanocrystalline itraconazole composition as a result of the higher supersaturation, which increased the permeation [22].

### 3.4. Ocular Drug Delivery

Drug delivery to eye tissues is particularly problematic because of generally poor drug bioavailability, drug instability, short residence time, poor drug solubility, a low amount of aqueous humor, and the loss of the drug with tears. Nanocrystals (nanosuspensions) can enhance ocular drug permeation, favor controlled release, and promote targeting [19,119], also guaranteeing fewer or more attenuated side effects than traditional formulations [120]. 

An example is offered by the formulation of nanosuspensions of three practically insoluble glucocorticoid drugs (hydrocortisone, prednisolone, and dexamethasone). They showed an enhanced rate and extent of ophthalmic drug absorption, and an increased intensity in the action of the drug. An increase in bioavailability has the important advantage of reducing the risks of adverse side effects associated with large doses of these drugs, such as cataracts, glaucoma, and optic nerve injury [121]. 

The bioavailability of dexamethasone acetate was improved by increasing the saturation solubility and the residence time in the eye of an ophthalmic cationic nanocrystal formulation. The saturation solubility increased due to the nanosize of the crystals, while the residence time improved due to increased mucoadhesion by the cationic charge [31].

Nanocrystal suspensions of brinzolamide, a poorly soluble drug, were prepared to reduce the intraocular pressure. At both tested pHs, 7.4 and 4.5, 100% of the drug dissolved in one minute. The lowering of intraocular pressure was investigated in vivo in rats and proved to be particularly effective [13,122].

### 3.5. Dermal Drug Delivery

Since 2006, with the first use of nanocrystals to increase the bioavailability of functionals upon skin delivery [123], many studies have sought to exploit the nanocrystal formulations of poorly soluble drugs to increase their bioavailability by skin delivery [18,23]. 

There are several major advantages to the nanocrystal dermal administration route. The higher particle surface of nanocrystals can enable increases in particle spreading and adhesiveness. Also, such formulations offer an increased dissolution rate and increased molecule flow due to improved saturation solubility [8]. 

Vidlářová et al. [21] demonstrated that nanosuspensions can penetrate through the skin and accumulate in the viable epidermis, and investigated the mechanism of penetration of curcumin nanocrystals through the skin. Nanocrystals produced through bead milling followed by high-pressure homogenization were formulated as low viscous nanosuspension at decreasing concentrations (2.0%, 0.2%, 0.02%, 0.002%) and compared to nanocrystals in viscous hydroxypropyl cellulose gel. The authors found that low viscosity favors skin penetration and observed no significant differences in penetration profiles for formulations with higher nanocrystal concentration, while significantly lower penetration was exhibited for the 0.002% concentration. They concluded that the concentration gradient cannot totally explain the driving force for the skin penetration, in contrast to the generally accepted understanding. Vidlářová et al. proposed other mechanisms to explain their findings. Perhaps the nanosuspensions have higher kinetic saturation solubility than the nanocrystals in the gel form. Or, it may be that the nanocrystal concentration is able to adequately cover the skin surface, or it may be due to the large crystal surface area in contact with the lipid film of the stratum corneum. Nanocrystal skin penetration is therefore still debated, particularly because the authors frequently do not further investigate this aspect in their studies.

For example, the importance of saturation solubility was highlighted by Colombo et al. [8]. The saturation solubility and dissolution rate were determined for nanocrystals of dexamethasone, ibuprofen, and tacrolimus. Nanocrystals prepared by bead milling (approximately 300 nm) exhibited far higher saturation solubility compared to non-milled particles (1.0 μm), and nanomilling increased drug dissolution rates, particularly for ibuprofen. In this study, the authors evaluated the effect of nanosize on the saturation solubility, but did not correlate the saturation solubility with transdermal penetration, or evaluate the potential of the absorption of nanocrystals through the skin. 

In another study, nanocrystals of the flavonoid apigenin produced by a combination of bead milling and subsequent high-pressure homogenization were formulated into a hydrogel for topical application. The nanometric particle size positively affected the ability of particles to coat the skin. The antioxidant capacity of apigenin was demonstrated by evaluating its antiradical scavenging activity. The fast release profile from nanometric range particles was also proven, but the effect on drug absorption was only supposed from previous data. No evaluation of the possible penetration of nanoparticles through the skin was provided in this study [92]. 

Interestingly, Zhai et al. [23] reported a mechanism for nanocrystal penetration through hair follicles. Nanocrystals in suspension demonstrated good saturation solubility and thus good concentration gradient, which allowed increased penetration and accumulation in hair follicles. 

## 4. Physicochemical Stability

While designing, developing, and optimizing a nanocrystal process at the industrial level, it is necessary to address several issues, particularly those related to instability problems. 

Particle agglomeration and amorphization/crystallization, which are major issues for drug stability, are discussed in the following section. Table 2 summarizes the issues related to the development and production of stable nanocrystals.

### 4.1. Particle Agglomeration and Stabilization

The excess in Gibbs’s free energy that is typical of particles of nanodimensions explains their tendency to agglomerate and/or aggregate to a less energetic state, and agglomeration/aggregation is a way to increase particle size, reduce surface energy, and minimize total energy [46]. The interaction between particles was described by Derjaguin and Landau [130] and then by Verwey and Overbeek [131]: their theory explaining the stability of colloidal suspension was termed the DLVO theory, which is an acronym formed by the first letter from each scientist’s last name.

It quantitatively describes the forces interacting between charged surfaces and a liquid medium, and it combines the effects of van der Waals attraction and electrostatic interactions (attraction or repulsion). The high surface energy produced during the preparation of nanocrystals or nanosuspension generally requests the use of stabilizers to prevent agglomeration [132,133].

Stabilizers thoroughly coat nanoparticles and prevent agglomeration, providing ionic or steric barriers [19]. To stabilize drug nanosuspension, researchers exploit amphiphilic excipients (frequently surfactants) or polymers with hydrophilic and hydrophobic domains that favor the interaction between particles and wetting liquid. Ionic surfactants and charged polymers can prevent aggregation by electrostatic repulsion, while non-ionic surfactants and non-charged polymers can form a steric barrier around the particles, preventing aggregation. 

An exhaustive list of stabilizers that can be used in drug nanosuspensions/nanoparticles stabilization was provided by Van Eerdenbrugh et al. [132]. 

Several examples about the tendency to particle agglomeration and the use of stabilizers to prevent particle agglomeration are provided in Table 3.

A study to limit particle aggregation was conducted on rutin nanocrystals [16] prepared as dried powder by lyophilization in the presence of 0.2% *W/W*% of sodium dodecyl sulfate (SDS) as an ionic surfactant. Particle sizes and physical stability were determined by photon correlation spectroscopy (PCS) and laser diffractometry (LD) immediately after preparation and after redispersion. Light microscope magnification by 1000 detected the nanosuspensions effectively. The authors were able to confirm that lyophilized nanoparticles redispersed in water without the aggregation due to electrostatic repulsion that otherwise would have impeded adequate particle dissolution. 

Spironolactone was coated with different non-ionic stabilizers such as poloxamers 407 and 188, hydroxypropyl methylcellulose, and ionic stabilizers such as sodium hydroxycholate. The oral bioavailability of spironolactone stabilized with sodium hydroxycholate was much lower than that of the other formulations. Actually, the ionic stabilizer revealed instability in gastric juice, inducing the aggregation of drug crystals [134]. In a study of polymeric stabilizers for drug nanocrystal dispersions, Choi et al. evaluated drug particle size by laser light scattering analysis, and found that it decreased with time to a limited value (steady-state) that depended on polymeric stabilizers. To understand this phenomenon better, they measured the contact angle between seven drugs (ibuprofen, naproxen, prednisolone acetate, nifedipine, hydrocortisone acetate, itraconazole, and anthracene) and two polymers (hydroxypropyl cellulose, or HPC, and polyvinylpyrrolidone, or PVP), and found that the surface energy of drugs and polymers was an important factor in influencing the steady state. They theorized that a correlation could be established by the chemical interaction between the drug and the stabilizer, in particular, that the stabilizer could be absorbed on the drug nanocrystal surface, thus providing a steric stabilization effect [135].

In another study, indomethacin and itraconazole were wet milled in the presence of four types of stabilizers (poloxamer 188, poloxamer 407, polysorbate 80, and PEG). The mean particle size and the polydispersity index (PI) were determined by photon correlation spectroscopy (PCS). They found that the lower the PI value, the more monodisperse the particles were. The morphological evaluation of particles was determined by transmission electron microscopy (TEM). These techniques were selected to reveal the particle size reduction over time and the agglomeration tendency of the particles. The amphiphilic block copolymers (poloxamer 188 and 407) appeared to stabilize the nanoparticles more efficiently than the low molecular weight surfactant (polysorbate 80). Particle size reduction and stability were affected negatively by high viscosity solutions, such as poloxamer 188 for itraconazole, and PEG for indomethacin and itraconazole. The stabilizers did not affect the crystalline state of the drugs, as proven by differential scanning calorimetry (DSC) and X-Ray powder diffraction (XRPD) [136]. 

Quercetin nanocrystals prepared by three methods, namely, high-pressure homogenization, bead milling, and cavi-precipitation, were characterized for their physicochemical properties, and compared for stability after storage in a refrigerator (4 ± 2 °C), at room temperature (25 ± 2 °C), and in an oven (40 ± 2 °C) for 180 days. All of the nanocrystals produced by the three methods were crystalline, as confirmed by X-ray diffraction study. Nanocrystals produced by cavi-precipitation showed lower stability than those produced by the other two methods. In particular, recrystallization and agglomeration were responsible for the increase in particle size due to agglomeration tendency, according to the Ostwald ripening phenomenon. The authors explained that this instability was due to competition between the solvent (ethanol) used for the cavi-precipitation and the surfactant (Tween 80), which should have prevented particle agglomeration. The partial dehydration of the surfactant due to the presence of ethanol provoked the particle agglomeration (as confirmed by PCS, LD, scanning electron micoscopy, or SEM, and the measurement of zeta potential by dynamic light scattering, or DLS) [87]. 

Nanocrystals of apigenin, a low water soluble flavonoid, were produced by a combination of bead milling and subsequent high-pressure homogenization [92]. An apigenin macrosuspension was prepared in the presence of Plantacare^®^ 2000 (alkyl polyglucoside) under high shear mixing. Dispersion was treated under bead milling and then under high-pressure homogenization. This combination process made it possible to reduce particle size to approximately 150 nm, with a low polydispersity index, as proven by PCS and LD. The X-ray powder diffractometry showed that apigenin did not undergo amorphization, and that the process avoided the particle agglomeration or any Ostwald ripening instability, guaranteeing high formulation physicochemical stability. The combination of low energy (bead milling) and high energy (high pressure homogenization) processes together with the use of a stabilizer could explain the high physicochemical stability of apigenin nanocrystals.

Hesperetin flavonoid nanosuspensions were prepared by high-pressure homogenization. Poloxamer 188, Inutec^®^ SP1, Tween 80, or Plantacare^®^ 2000 were used as stabilizers to prevent particle agglomeration. The ability to reduce particle size, detected by PCS, LD, and DLS, was approximately the same in the presence of all of the stabilizers (approximately 300 nm), and no statistically significant differences in polydispersity index could be highlighted. Slight aggregation was observed in the presence of Tween 80 (final particles were of 350 mean particle size). Inutec and Plantacare proved to be the best stabilizers, which were able to prevent any change in particle size and zeta potential even after two years of stability study, while the worst cases were those obtained using poloxamer and Tween 80 [90]. The explanation was found in the differences in the viscosity of the system: highly viscous systems prevent aggregation and stabilize nanosuspensions.

These same conclusions were reached in another study in which nanosuspensions of resveratrol were produced by high-pressure homogenization in the presence of the same stabilizers used in the previous study (Poloxamer 188, Inutec SP1, Tween 80, or Plantacare^®^ 2000). Also in this study, the best stabilization was achieved in the presence of 1% of Plantacare or Inutec [88].

Nanocrystals of caffeine, a medium soluble drug, lead to pronounced crystal growth [23]. Nanocrystals were prepared by pearl milling in the presence of stabilizers such as PVP 40, Carbopol^®^ 981, or Tween^®^ 80, and in the presence of water or ethanol as suspender liquids. In the study, it was proven that crystal growth revealed by PCS and light microscopy may be affected by several factors, such as the suspender liquid, the surfactant, and the steric stabilizer. It is possible that the latter two are absorbed into the crystal surface to different extents, thus affecting crystal growth. In this study, Carbopol^®^ 981 was revealed as the best stabilizer even in its not neutralized form. In this case, the polymer can protonate and charge the newly created crystal surfaces of caffeine, which actually possesses several protonable groups, such as carbonyl or imine groups. The consequence is stabilization via electrostatic repulsion. 

### 4.2. Amorphization and Crystallization

One of the classic approaches for enhancing drug bioavailability is the conversion of the crystalline drug to its amorphous form, because amorphous drugs are markedly more soluble than their crystalline counterparts [45,137]. The thermodynamic and kinetic properties of the amorphous state, such as an excess of enthalpy, entropy, and free energy, explain the highest solubility and dissolution rate of amorphous forms with the respect to crystalline one [35]. 

In general, the processes used to amorphize solids are fast solidification, solidification from melt, drying procedures (such as freeze-drying or spray-drying), grinding, and compression [125,126,128,129].

Specifically, the most widely applied and developed pharmaceutical technologies to promote drug amorphization are solid dispersions and nanocrystal technologies. They share the same objective and similar issues, but apply different technologies [4,138]. 

In the past, even though pharmaceutical industries recognized the advantage of amorphous formulations for increasing drug bioavailability, they did not focus on the development and marketing of amorphous formulations because of problems related to physicochemical stability and the modification of the drug bioavailability during the drug product shelf life. More recently, though, the emergence of innovative strategies to stabilize amorphous drugs has driven an increase in patented amorphous formulations and new FDA-approved amorphous drug products [4]. 

In line with this trend, there has been an increase in the number of studies seeking to produce amorphous nanocrystals. 

In general, the application of high pressures during the production of nanosuspensions promotes solid amorphization, while low-energy processes favor the achievement of completely crystalline structures [26,139].

Nanocrystals of nicergoline of a mean particle diameter of nearly 700–800 nm were prepared by a nanospray drying method [51]. Spherical nanocrystals were proven to be amorphous by DSC and XRPD, and stability tests revealed the good physical stability of the amorphous nanocrystals stored at 3 °C for at least one year, confirming the results of a previous study that demonstrated that amorphous nicergoline had high physicochemical stability [33]. The observation that nanocrystals have a more rapid dissolution rate than native and coarse particles was explained by the particle dimensions and amorphous physical state of the nanocrystals.

The same nanospray drying procedure was applied to indomethacin (IDM) [64], along with two other methods: wet milling followed by lyophilization and cryomilling. Under the three methods, it was possible to recover pure particles of a mean particle diameter ranging between 500–800 nm. During these three treatments, IDM underwent physicochemical modifications. Particles obtained under nanospray drying underwent partial amorphization and consequently crystallization under the metastable polymorphic form α. Thus, all of the batches produced through this method were a mixture of amorphous and polymorphic forms α (the metastable one) and γ (the native and stable polymorphic form). IDM treated by the other two methods exhibited a different tendency to amorphization, but at the end, only the γ form was present. The fastest intrinsic dissolution rates were observed for the batch prepared under nanospray drying and the one prepared after cryomilling for 40 min. These results were influenced by the crystalline form and by a decrease in particle size, which also influenced the particle dissolution behavior. 

Cryomilling was also applied to glibenclamide [93], and particles of nearly 500 nm in mean diameter were obtained. A significant decrease in crystallinity degree was revealed by DSC and XRPD. Both the decrease in particle size and crystallinity degree concurred to improve the particle dissolution rate. 

Trasi and Byrn [47] studied the physicochemical behavior under cryomilling of six different compounds with different properties to obtain an amorphous solid. Ketoconazole, ursodiol, indomethacin, griseofulvin, carbamazepine, and piroxicam were investigated by DSC, XRPD, and hot-stage microscopy, which indicated that all of the drugs underwent progressive amorphization during cryomilling.

Cryomilling does not always cause amorphization or changes in the crystallinity degree of drugs. In the case of both phenytoin and ibuprofen, no changes in physical state were observed by DSC and XRPD [127]. However, an important tendency to particle agglomeration was highlighted by PCS. 

On the other hand, Kayaert and Van den Mooter [140] proved that milling cannot be considered the main cause of amorphization for nanosuspensions, and that water present in the nanosuspensions can act as a plasticizer that triggers recrystallization. In their study, they selected cinnarizine and naproxen as model drugs to produce nanosuspensions by ball milling, stabilized with hydroxypropyl methyl cellulose (HPMC 2910). Cinnarizine was selected as a weak hydrophobic base, and naproxen was selected as a weak acid. They explained that the cause of amorphization can be found in the interplay between drug and stabilizer after drying. If a drug is soluble in the stabilizer in the solid state, an amorphous solid dispersion is formed at the interface. 

Dry milling and wet milling (in the presence of water) can give different results concerning the drug amorphization tendency, as demonstrated by two separate studies. In the first, the dry milling of indomethacin at both cryogenic and room temperature resulted in an amorphous form, whether a stabilizer was used or not [124,141]. In the second, indomethacin was subjected to wet milling under high-pressure homogenization in the presence of PVP K25 and poloxamer 407 as suspension stabilizers. As proved by infrared spectroscopy (IR) and modulated differential scanning calorimetry (MTDSC), indomethacin was only partially amorphized, and the amorphous form was only present on the surface and for an amount lower than 1%. The authors concluded that the amorphization was prevented in the presence of water, which inhibits the drug amorphization during wet milling [91]. 

### 4.3. Particle Surface Modification

This review has included several examples of the use of excipients as stabilizers, but it should be noted that the addition of stabilizers on the particle surface not only acts on the physical stabilization of particles, it may also provide additional properties to the nanocrystals, modifying their bioavailability and pharmacological activity (Table 3).

Arginine, albumin, leucin, vitamin E, polyethylene glycol succinate (TPGS), lecithin, hydroxypropyl methyl cellulose, and sodium cholic acid, provide additional favorably biological properties to nanocrystals.

For example, the current intravenous therapy with nimodipine is associated with great pain and local irritation, and in some cases phlebitis during infusion, due to the presence of the high quantity of ethanol that is necessary to dissolve the poorly soluble drug. Nimodipine was thus formulated as a nanosuspension in the presence of poloxamer 188 and sodium cholic acid. In addition to the increased dissolution rate, this nanosuspension was proven to be less irritant and cause less phlebitis than ethanol products [142].

In another study, it was demonstrated that the hydrophobicity of copolymers is a crucial parameter in the preparation of drug nanocrystals by wet comminution. Actually, amino acid copolymers containing lysine as hydrophilic segments, and phenylalanine or leucine as their hydrophobic segments, were used to produce stable nanocrystals (200–300 nm) in water [143].

Paclitaxel nanocrystals were stabilized by serum protein transferrin in a non-covalent manner. In vivo anti-tumor efficacy studies demonstrate a higher tumor inhibition rate of 45.1% for paclitaxel–transferrin formulation compared to 28.8% for paclitaxel nanosuspension treatment alone, and paclitaxel–transferrin formulation showed a lower level of toxicity. Thus, paclitaxel nanocrystals can be formulated in the presence of serum protein as a promising drug delivery model for anticancer therapy [144].

The residence time of nanocrystals in the gastrointestinal tract can be increased by improving the adhesiveness of nanocrystals to lumen with the incorporation of mucoadhesive polymers [145]. A number of studies describe the preparation of mucoadhesive nanocrystal formulations. Among them, Jacobs et al. [101] reported the formulation of a mucoadhesive hydrogel of different Carbopols containing a nanosuspension of buparvaquone, which is an antibiotic frequently used in AIDS patients. The oral bioavailability of buparvaquone is limited, and thus the mucoadhesive hydrogel promotes a prolonged retention time of the drug nanosuspension in the infected gastrointestinal tract. 

In another case, orally administered nanosuspensions were modified on the surface through the absorption of mucoadhesive polymers such as chitosan and Carbopol, which can increase the adhesion to the gut wall [146]. A mucoadhesive nanosuspension of hydrocortisone acetate in the presence of poloxamer 188 and chitosan chloride was produced by high-pressure homogenization as layering dispersion in a fluidized bed process, followed by the application of an enteric coating to achieve controlled drug release. Pellets containing drug nanocrystals exhibited accelerated dissolution velocity and increased drug release compared to a reference formulation of microparticles [28].

Budesonide, a poorly water-soluble corticosteroid anti-inflammatory drug, was formulated as nanocrystals loaded into hyaluronic acid microparticles. Budesonide nanocrystals exhibited prolonged retention on the surface porcine tracheal tube thanks to the mucoadhesive properties of hyaluronic acid, which significantly improved the drug bioavailability [14]. Hyaluronic acid was used to coat paclitaxel nanocrystals to favor long circulation and tumor-targeting properties. In vivo studies confirmed the increase of nanocrystals’ blood circulation time. In addition, the interaction of nanocrystals coated with hyaluronic acid with CD44 receptors was confirmed together with the role in enhancing the uptake and efficacy of paclitaxel against cancer cells. 

When nanoparticles are administered by injection, the immune system recognizes them as foreign particles, and they are immediately opsonized by proteins and enzymes circulating in the blood to be taken up by phagocyte cells [10,147]. Thus, nanoparticles are distributed to the tissues and body compartments (particularly the reticulum endothelial system) according to their size, zeta potential, and composition. This phenomenon is termed passive targeting, and is exploited to target drugs to specific compartments [39,105]. The PEGylation of nanocrystals can prolong their circulation time, limiting opsonization and improving the therapeutic effect of the loaded drug. Several examples are offered in the case of paclitaxel [11,81,106,148]. In particular, PEGylated nanocrystals of paclitaxel (PTX) were prepared using antisolvent precipitation augmented by probe sonication. The PEG molecules covered the surface of nanocrystals with an 11.54-nm fixed aqueous layer thickness (FALT). PEGylated nanocrystals showed significant tumor inhibition in breast cancer xenografted mice [81].

Coating nanocrystals with surfactants can modify the permeation at the blood-brain barrier (BBB), allowing the barrier crossing and access to treat brain diseases. 

Atovaquone can be safely and effectively used against *T. gondii* in vitro to treat toxoplasmic encephalitis, but the oral micronized solution shows poor bioavailability [149]. In vivo studies confirmed the capacity of nanosuspensions coated with sodium dodecyl sulfate to cross the blood–brain barrier and permit the treatment of toxoplasmic encephalitis and other cerebral diseases.

Nanosuspensions of amphotericin B were produced by high-pressure homogenization in the presence of different surfactants such as Tween 80, Tween 20, sodium cholate, Pluronic F127, Pluronic F68, which were all approved for intravenous injection. The results indicated that nanosuspensions coated with polysorbate 80 and sodium cholate increased drug brain delivery and inhibited the parasite in vitro. The in vivo results were less satisfactory, which was probably because of the severe and rapid pathological effects of the parasite once it reached the brain [89].

Active targeting, in contrast, is achieved by coating the particles with specific molecules that are able to interact with receptors or biological substrates in general [18].

Gold nanocrystals (“nanorods”) have the advantage of accumulating in the tumor cells. They were stabilized with a layer of polyethylene glycols (PEGs) and covalently conjugated to tumor-targeting peptides: (i) a single-chain variable fragment (ScFv) peptide that recognizes the epidermal growth factor receptor (EGFR); (ii) an amino terminal fragment (ATF) peptide that recognizes the urokinase plasminogen activator receptor (uPAR); and (iii) a cyclic RGD peptides that recognizes the A_v_B_3_ integrin receptor. In vivo studies confirmed that they alter the intracellular and extracellular nanoparticle distributions, and that the preferred administration route is intratumoral injection instead of intravenous injection [39]. In another study, serum albumin surface modified nanosuspensions of nevirapine, a non-nucleoside reverse transcriptase inhibitor, were prepared by high-pressure homogenization technique and stabilized with a surfactant solution. The biodistribution studies showed a higher accumulation of the nanosuspension in various organs of rat compared to the plain drug solution administered intravenously. Macrophage uptake showed the ability of coated nanosuspension to target the intracellular compartments, while higher drug levels were identified in the spleen, liver, and thymus without added cytotoxicity. The surface modification of the nevirapine nanosuspensions with serum albumin favored the blood–brain barrier crossing and accumulation in the brain for more than 24 h [20]. Nanocrystals of docetaxel with a size <200 nm, negative zeta potential, and 98% drug content were prepared in the presence of a novel polymeric conjugate comprising chondroitin sulfate A and polyethylene glycol. The glycoconjugate favors stability, stealth effects, and tumor-targeting via CD44 receptors to the drug. The enhanced permeability, the retention effect, and the endocytosis receptor that were mediated permit the penetration of the docetaxel system into the cells of tumor mass. Excellent tumor inhibition and less unwanted toxicity were demonstrated in vitro and in vivo [150].

## 5. Conclusions

In recent decades, nanocrystal formulations have emerged as a very interesting approach to improve the bioavailability of poorly soluble drugs. Initially, this was the principal interest in nanocrystals, but more recently, their application has evolved toward other goals such as sustained release, modified drug delivery, and drug targeting. 

Thus, nanocrystals are now used to achieve pharmacological objectives similar to those for which polymeric nanoparticles have been employed for several decades. Similar to polymeric nanoparticles, they also have negative tendencies to instability, as seen in aggregation or a change in the solid state of the drug, which must be addressed wisely in preparing effective formulations. 

The full comprehension of these phenomena and methods for stabilization are fundamental in the design and production of nanosystems. Surface stabilizers hold the crucial role of promoting the physical stabilization of nanocrystals, and even their biological functionalization. The choice of the appropriate stabilizer is rather difficult, and this review tried to offer many examples about the use of several classes of stabilizers in achieving the different goals.

Nonetheless, in spite of the large literature on the positive results concerning the improvement of drug nanocrystal bioavailability, the number of approved drug products is still limited. The research and development (R&D) and the pharmaceutical industry now have to concentrate their efforts to optimize scalable processes and formulations, and allow for an appropriate physicochemical and biological stability during the shelf life of the drug product. 

## Figures and Tables

**Table 1:** Main technologies applied for the production of nanocrystals (nanosuspensions, nanocomposites). References were approximately focused on the last 10 years.

Technique			Drug	References
Bottom-Up	Evaporation methods	Spray-drying	Budesonide, nicergolide, indomethacin, cinnarizine, griseofulvin, mebendazole	[14,33,51,64,65]
		Freeze-drying	Fenofibrate	[12]
		Vacuum-drying	Itraconazole, naproxen, sofalcone, colostazol	[66,67]
		Aerosol flow reactor	Beclomethasone dipropionate	[68]
		Electrospraying	Insulin	[69]
		Fluid bed coating/granulation/drying	Hydrocortisone acetate, ketoconazole, griseofulvin, phenylbutazone	[28,70,71]
		Wet-casting drying	Griseofulvin, naproxen, fenofibrate,	[72,73,74,75,76]
		Nanoextrusion	Phenytoin, efavirenz, griseofulvin	[77,78,79,80]
	Precipitation methods	Solvent–antisolvent precipitation	Paclitaxel	[81]
		High gravity precipitation	Cefuroxime axetil, cephradine, azithromycin, danazol, Salbutamol Sulphate	[82,83,84,85]
		Flash Precipitation	Cyclosporine A	[86]
		Sonoprecipitation	Fenofibrate, paclitaxel	[12,81]
		Supercritical Fluid	Apigenin	[9]
Top-Down	High pressure homogenization	Microfluidification	Bexarotene	[7]
		Piston gap homogenization	Nimodipine, lutein, asulacrine, baicalein, apigenin, quercetin, hesperetin, resveratrol, indomethacin, hydrocortisone acetate, nevirapine, amphotericin B	[9,10,17,20,24,28,87,88,89,90,91]
	Bead milling		Apigenin, dexamethasone, ibuprofen, tacrolimus, quercetin	[8,87,92]
	Cryomilling		Indomethacin, glibenclamide, ketoconazole, ursodiol, indomethacin, griseofulvin, carbamazepine, piroxicam	[47,64,93]

**Table 2:** Summarization of the main instabilities affecting nanocrystals (nanosuspensions).

Main Instability	Techniques Provoking the Instability	References
particle aggregation	Wet comminution	[8,9,16,17,20,21,24,28,64,87,92]
	Lyophilization	
	High-pressure homogenization	
	Bead milling	
	Cavi-precipitation	
	Dehydration of the surfactant	
amorphization	Spray-drying	[10,14,16,22,47,64,93,124,125,126,127,128,129]
	Lyophilization	
	Dry milling	
	Cryomilling	
	Wet milling	
crystallization	Antisolvent	[24,64]
	High-pressure homogenization	
	Nanospray drying underwent	

**Table 3:** Stabilization and functionalization of nanocrystals (nanosuspensions) by means of different stabilizers and surface agents.

Type of Nanocrystal Surface Modifier	Mechanism	Drug—Active Compound	Applied Technology	References
Ionic surfactants/charged polymers: sodium cholate, sodium deoxycholate, sodium lauryl sulfate, sodium dodecyl sulfate, sodium poly(ethylene imine), chitosan	Electrostatic repulsion (prevent aggregation)	Albendazole	Nanoprecipitation	[151]
			Sonication	
			High-pressure homogenization	
			High-speed homogenization	
			Milling	
		Spironolactone	Wet milling	[134]
			High-pressure homogenization	[152]
		Curcumin	High-speed homogenization	[153]
			High-pressure homogenization	[154]
			Nanoprecipitation method	[155]
		Nitrendipine	Precipitation + high-pressure homogenization	[156]
		Rutin	High-pressure homogenization	[16]
Non ionic surfactant/polymers: celluloses, polyvinyl alcohol, polyvinyl pyrrolidone, polysorbates, pluronic, poloxamers, triblock-copolymers of polyoxyethylene and polyoxypropylene, hydroxypropyl methylcellulose	Steric barrier against aggregation (prevent aggregation)	Albendazole	Nanoprecipitation	[151]
			Sonication	
			High-pressure homogenization	
			High-speed homogenization	
			Milling	
			Antisolvent precipitation method	[157]
		Nitrendipine	Precipitation + high-pressure homogenization	[158,159]
		Ibuprofen	Wet comminution	[135]
		Naproxen		
		Prednisolone acetate		
		Hydrocortisone acetate		
		Anthracene		
		Itraconazole	Wet comminution/Wet milling	[135,136]
		Indomethacin	Wet milling	[136]
		Quercetin	High-pressure homogenization	[87,160]
			Bead milling	
			Cavi-precipitation	
		Apigenin	Bead milling + high-pressure homogenization	[92]
		Hesperetin	High-pressure homogenization	[90]
		Resveratrol	High-pressure homogenization	[88,161]
			Precipitation	[162]
		Caffeine	Pearl milling	[23]
		Curcumin	High-speed homogenization	[153]
			Wet milling	[163]
			High-pressure homogenization	[154]
			Nanoprecipitation	[155]
		Dexamethasone	Wet milling	[164]
		Diclofenac	Wet milling	[165,166]
		Pyrimethamine	Nanoprecipitation + high-pressure homogenization	[167]
		Nifedipine	High-pressure homogenization	[168,169]
		Spironolactone	Wet milling	[134]
Alkyl polyglucoside (Plantacare^®^ 2000), hydroxypropyl methyl cellulose (HPMC 2910), polyvinyl pyrrolidone, and poloxamer	Formation of an amorphous solid dispersion at the interface (prevent amorphization)	Apigenin	Bead milling + high-pressure homogenization	[92]
		Cinnarizine and naproxen	Ball milling	[140]
		Indomethacin	Dry milling	[124]
			Wet milling	[91]
		Fenofibrate	Milling	[170]
Arginine, amphiphilic amino acid copolymers (albumin, leucin), vitamin E, polyethylene glycol succinate (TPGS), lecithin, hydroxypropyl methyl cellulose, sodium cholic acid	Biological active providing additional functions to nanocrystals (promotion of a stable formulation)	Nimodipine	High-pressure homogenization	[142]
		Naproxen	Wet comminution	[143,171]
		Amoitone B	High-pressure homogenization	[40]
		Prednisolone, carbamazepine, itraconazole, baicalin, cyclosporine	High-pressure homogenization	[146]
		Paclitaxel	Antisolvent precipitation + sonication	[144]
		Curcumin	High-pressure homogenization	[153]
		Caffeine	Pearl milling	[23]
Chitosan (amino group), Carbopol	Mucoadhesion (promote absorption)	Hydrocortisone acetate	High-pressure homogenization	[28,146]
		Buparvaquone	High-pressure homogenization	[101,146]
Cetylpyridinium chloride (CPC) and benzalkonium chloride (BAC)	Mucoadhesion by the positively charged nanocrystal formulation	Dexamethasone acetate	Wet bead milling	[31]
Hyaluronic acid	Mucoadhesion by gelation (promote absorption)	Budesonide	Wet ball milling	[14]
	Improving long circulation and interaction with specific receptors	Paclitaxel	High-pressure homogenization	[37]
Polyethylene glycol (PEG)	Improving biological stability	Nevirapine	High-pressure homogenization	[20]
		Pioglitazone	Precipitation	[172]
		Piroxicam	High-pressure homogenisation	[173]
		Meloxicam	High-pressure homogenization	[174]
		Itraconazole	Nanomilling	[175]
		Paracetamol	Precipitation	[176]
		Camptothecin	Three-phase nanoparticle engineering technology (3PNET)	[106]
		Paclitaxel	Antisolvent precipitation + sonication	[81]
			Wet milling	[148]
			Three-phase nanoparticle engineering technology (3PNET)	[106]
			Antisolvent precipitation	[11]
Surfactants: sodium dodecyl sulfate, polysorbate, sodium cholate, d-α-tocopheryl polyethylene glycol 1000 succinate	Modify the permeation at the blood–brain barrier (promote targeting)	Atovaquone	High-pressure homogenization	[149]
		20(*S*)-protopanaxadiol	Antisolvent precipitation	[177]
		Puerarin	Ultrasonic	[178]
		Amphotericin B	High-pressure homogenization	[89]
Single-chain variable fragment (ScFv) peptide, amino terminal fragment (ATF) peptide, cyclic RGD peptides, folate ligand (F127), serum albumin, dextran, Crystal lattice, Apo A-I and Apo A-IV, folate-conjugated polydopamine (PFA), chondroitin sulfate A, chimeric DNA molecules, 3-chloro-2-hydroxy-1-propanesulfonic, acid sodium salt hydrate (CPSA) and 4-sulfophenyl isothiocyanate sodium salt monohydrate (4-SPITC), acid folic, apolipoprotein E	Promote the specific interaction with the biological substrate (promote targeting)	Gold nanorods	Precipitation	[39]
		Baicalin	High-pressure homogenization	[179]
		Paclitaxel and camptothecin	3-Phase nanoparticle engineering technology (3 PNET)	[106]
		Nevirapine	Cold high-pressure homogenization	[20]
		Paclitaxel	Antisolvent process: sonication	[24,180]
			High-pressure homogenization	[39]
		Hydroxycamptothecin	Antisolvent process	[107]
		Docetaxel	High-pressure homogenizer + heat exchanger	[150]
		Gemcitabine	Nanoprecipitation	[181]
		Naproxen and paclitaxel	Nano-comminution	[182]
		Camptothecin	Precipitation	[183]

## References

[1] Loftsson T., Brewster M.E. (2010). Pharmaceutical applications of cyclodextrins: Basic science and product development. J. Pharmacy Pharm..

[2] Keck C.M., Müller R.H. (2006). Drug nanocrystals of poorly soluble drugs produced by high pressure homogenisation. Eur. J. Pharm. Biopharm..

[3] Aguiar G.P.S., Marcon M., Mocelin R., Herrmann A.P., Chaves L.M., Piato A.L., Lanza M., Oliveira J.V. (2017). Micronization of n-acetylcysteine by supercritical fluid: Evaluation of in vitro and in vivo biological activity. J. Supercrit. Fluids.

[4] Jermain S.V., Brough C., Williams R.O. (2018). Amorphous solid dispersions and nanocrystal technologies for poorly water-soluble drug delivery—An update. Int. J. Pharm..

[5] Junghanns J.-U.A., Müller R.H. (2008). Nanocrystal technology, drug delivery and clinical applications. Int. J. Nanomed..

[6] Peltonen L., Hirvonen J. (2018). Drug nanocrystals-versatile option for formulation of poorly soluble materials. Int. J. Pharm..

[7] Chen L., Wang Y., Zhang J., Hao L., Guo H., Lou H., Zhang D. (2014). Bexarotene nanocrystal—Oral and parenteral formulation development, characterization and pharmacokinetic evaluation. Eur. J. Pharm. Biopharm..

[8] Colombo M., Staufenbiel S., Rühl E., Bodmeier R. (2017). In situ determination of the saturation solubility of nanocrystals of poorly soluble drugs for dermal application. Int. J. Pharm..

[9] Fu Q., Sun J., Ai X., Zhang P., Li M., Wang Y., Liu X., Sun Y., Sui X., Sun L. (2013). Nimodipine nanocrystals for oral bioavailability improvement: Role of mesenteric lymph transport in the oral absorption. Int. J. Pharm..

[10] Ganta S., Paxton J.W., Baguley B.C., Garg S. (2009). Formulation and pharmacokinetic evaluation of an asulacrine nanocrystalline suspension for intravenous delivery. Int. J. Pharm..

[11] Gao W., Chen Y., Thompson D.H., Park K., Li T. (2016). Impact of surfactant treatment of paclitaxel nanocrystals on biodistribution and tumor accumulation in tumor-bearing mice. J. Control. Release.

[12] Ige P.P., Baria R.K., Gattani S.G. (2013). Fabrication of fenofibrate nanocrystals by probe sonication method for enhancement of dissolution rate and oral bioavailability. Colloids Surf. B Biointerfaces.

[13] Khan M.S., Vishakante G.D., Bathool A. (2013). Development and characterization of pilocarpine loaded eudragit nanosuspensions for ocular drug delivery. J. Biomed. Nanotechnol..

[14] Liu T., Han M., Tian F., Cun D., Rantanen J., Yang M. (2018). Budesonide nanocrystal-loaded hyaluronic acid microparticles for inhalation: In vitro and in vivo evaluation. Carbohydr. Polym..

[15] Liu Y., Huang L., Liu F. (2010). Paclitaxel nanocrystals for overcoming multidrug resistance in cancer. Mol. Pharm..

[16] Mauludin R., Müller R.H., Keck C.M. (2009). Development of an oral rutin nanocrystal formulation. Int. J. Pharm..

[17] Mitri K., Shegokar R., Gohla S., Anselmi C., Müller R.H. (2011). Lutein nanocrystals as antioxidant formulation for oral and dermal delivery. Int. J. Pharm..

[18] Muller R.H., Keck C.M. (2004). Challenges and solutions for the delivery of biotech drugs—A review of drug nanocrystal technology and lipid nanoparticles. J. Biotechnol..

[19] Patravale V., Date A.A., Kulkarni R. (2004). Nanosuspensions: A promising drug delivery strategy. J. Pharm. Pharmacol..

[20] Shegokar R., Singh K.K. (2011). Surface modified nevirapine nanosuspensions for viral reservoir targeting: In vitro and in vivo evaluation. Int. J. Pharm..

[21] Vidlářová L., Romero G.B., Hanuš J., Štěpánek F., Müller R.H. (2016). Nanocrystals for dermal penetration enhancement—Effect of concentration and underlying mechanisms using curcumin as model. Eur. J. Pharm. Biopharm..

[22] Yang W., Johnston K.P., Williams R.O. (2010). Comparison of bioavailability of amorphous versus crystalline itraconazole nanoparticles via pulmonary administration in rats. Eur. J. Pharm. Biopharm..

[23] Zhai X., Lademann J., Keck C.M., Müller R.H. (2014). Dermal nanocrystals from medium soluble actives–physical stability and stability affecting parameters. Eur. J. Pharm. Biopharm..

[24] Zhao R., Hollis C.P., Zhang H., Sun L., Gemeinhart R.A., Li T. (2011). Hybrid nanocrystals: Achieving concurrent therapeutic and bioimaging functionalities toward solid tumors. Mol. Pharm..

[25] Baba K., Pudavar H.E., Roy I., Ohulchanskyy T.Y., Chen Y., Pandey R.K., Prasad P.N. (2007). New method for delivering a hydrophobic drug for photodynamic therapy using pure nanocrystal form of the drug. Mol. Pharm..

[26] Liversidge G.G., Cundy K.C. (1995). Particle size reduction for improvement of oral bioavailability of hydrophobic drugs: I. Absolute oral bioavailability of nanocrystalline danazol in beagle dogs. Int. J. Pharm..

[27] Merisko-Liversidge E., Sarpotdar P., Bruno J., Hajj S., Wei L., Peltier N., Rake J., Shaw J., Pugh S., Polin L. (1996). Formulation and antitumor activity evaluation of nanocrystalline suspensions of poorly soluble anticancer drugs. Pharm. Res..

[28] Moschwitzer J., Muller R.H. (2006). Spray coated pellets as carrier system for mucoadhesive drug nanocrystals. Eur. J. Pharm. Biopharm..

[29] Yang C., Gao S., Dagnæs-Hansen F., Jakobsen M., Kjems J. (2017). Impact of peg chain length on the physical properties and bioactivity of pegylated chitosan/sirna nanoparticles in vitro and in vivo. ACS Appl. Mater. Interfaces.

[30] Gao L., Zhang D., Chen M., Zheng T., Wang S. (2007). Preparation and characterization of an oridonin nanosuspension for solubility and dissolution velocity enhancement. Drug Dev. Ind. Pharmacy.

[31] Romero G.B., Keck C.M., Müller R.H., Bou-Chacra N.A. (2016). Development of cationic nanocrystals for ocular delivery. Eur. J. Pharm. Biopharm..

[32] Shegokar R., Müller R.H. (2010). Nanocrystals: Industrially feasible multifunctional formulation technology for poorly soluble actives. Int. J. Pharm..

[33] Martena V., Censi R., Hoti E., Malaj L., Martino P.D. (2012). Physicochemical characterization of nicergoline and cabergoline in its amorphous state. J. Therm. Anal. Calorim..

[34] Sun J., Wang F., Sui Y., She Z., Zhai W., Wang C., Deng Y. (2012). Effect of particle size on solubility, dissolution rate, and oral bioavailability: Evaluation using coenzyme q10 as naked nanocrystals. Int. J. Nanomed..

[35] Yu L. (2001). Amorphous pharmaceutical solids: Preparation, characterization and stabilization. Adv. Drug Deliv. Rev..

[36] Wang T., Qi J., Ding N., Dong X., Zhao W., Lu Y., Wang C., Wu W. (2018). Tracking translocation of self-discriminating curcumin hybrid nanocrystals following intravenous delivery. In. J. Pharm..

[37] Sharma S., Singh J., Verma A., Teja V., Shukla R.P., Singh S., Sharma V., Konwar R., Mishra P. (2016). Hyaluronic acid anchored paclitaxel nanocrystals improves chemotherapeutic efficacy and inhibits lung metastasis in tumor-bearing rat model. RSC Adv..

[38] Lu Y., Li Y., Wu W. (2016). Injected nanocrystals for targeted drug delivery. Acta Pharm. Sin. B.

[39] Huang X., Peng X., Wang Y., Wang Y., Shin D.M., El-Sayed M.A., Nie S. (2010). A reexamination of active and passive tumor targeting by using rod-shaped gold nanocrystals and covalently conjugated peptide ligands. ACS Nano.

[40] Pawar V.K., Singh Y., Meher J.G., Gupta S., Chourasia M.K. (2014). Engineered nanocrystal technology: In-vivo fate, targeting and applications in drug delivery. J. Control. Release.

[41] Chaubal M.V. (2004). Application of formulation technologies in lead candidate selection and optimization. Drug Discov. Today.

[42] Shi W., Zeng H., Sahoo Y., Ohulchanskyy T.Y., Ding Y., Wang Z.L., Swihart M., Prasad P.N. (2006). A general approach to binary and ternary hybrid nanocrystals. Nano Lett..

[43] Hollis C.P., Weiss H.L., Leggas M., Evers B.M., Gemeinhart R.A., Li T. (2013). Biodistribution and bioimaging studies of hybrid paclitaxel nanocrystals: Lessons learned of the epr effect and image-guided drug delivery. J. Control. Release.

[44] Sailor M.J., Park J.H. (2012). Hybrid nanoparticles for detection and treatment of cancer. Adv. Mater..

[45] Hancock B.C., Parks M. (2000). What is the true solubility advantage for amorphous pharmaceuticals?. Pharm. Res..

[46] Merisko-Liversidge E., Liversidge G.G. (2011). Nanosizing for oral and parenteral drug delivery: A perspective on formulating poorly-water soluble compounds using wet media milling technology. Adv. Drug Deliv. Rev..

[47] Trasi N.S., Byrn S.R. (2012). Mechanically induced amorphization of drugs: A study of the thermal behavior of cryomilled compounds. AAPS PharmSciTech.

[48] Murray C.B., Kagan C.R., Bawendi M.G. (2000). Synthesis and characterization of monodisperse nanocrystals and close-packed nanocrystal asssemblies. Annu. Rev. Mater. Sci..

[49] Vishal P., Abhale V.N. (2016). Nanocrystal technology: A particle engineering formulation strategy for the poorly water soluble drugs. Int. J. Pharm..

[50] Pardeike J., Strohmeier D.M., Schrodl N., Voura C., Gruber M., Khinast J.G., Zimmer A. (2011). Nanosuspensions as advanced printing ink for accurate dosing of poorly soluble drugs in personalized medicines. Int. J. Pharm..

[51] Martena V., Censi R., Hoti E., Malaj L., Di Martino P. (2012). A new nanospray drying method for the preparation of nicergoline pure nanoparticles. J. Nanopart. Res..

[52] Bhakay A., Rahman M., Dave R.N., Bilgili E. (2018). Bioavailability enhancement of poorly water-soluble drugs via nanocomposites: Formulation processing aspects and challenges. Pharmaceutics.

[53] Chin W.W.L., Parmentier J., Widzinski M., Tan E.H., Gokhale R. (2014). A brief literature and patent review of nanosuspensions to a final drug product. J. Pharm. Sci..

[54] Malamatari M., Taylor K.M., Malamataris S., Douroumis D., Kachrimanis K. (2018). Pharmaceutical nanocrystals: Production by wet milling and applications. Drug Discov. Today.

[55] Arunkumar N., Deecaraman M., Rani C. (2014). Nanosuspension technology and its applications in drug delivery. Asian J. Pharm..

[56] Cooper E.R. (2010). Nanoparticles: A personal experience for formulating poorly water soluble drugs. J. Control. Release.

[57] Merisko-Liversidge E.M., Liversidge G.G. (2008). Drug nanoparticles: Formulating poorly water-soluble compounds. Toxicol. Pathol..

[58] Möschwitzer J. (2010). Particle size reduction technologies in the pharmaceutical development process. Am. Pharm. Rev..

[59] Möschwitzer J.P. (2013). Drug nanocrystals in the commercial pharmaceutical development process. Int. J. Pharm..

[60] Texter J. (2001). Precipitation and condensation of organic particles. J. Dispers. Sci. Technol..

[61] Fontana F., Figueiredo P., Zhang P., Hirvonen J.T., Liu D., Santos H.A. (2018). Production of pure drug nanocrystals and nano co-crystals by confinement methods. Adv. Drug Deliv. Rev..

[62] Arzi R.S., Sosnik A. (2018). Electrohydrodynamic atomization and spray-drying for the production of pure drug nanocrystals and co-crystals. Adv. Drug Deliv. Rev..

[63] Salazar J., Müller R.H., Möschwitzer J.P. (2014). Combinative particle size reduction technologies for the production of drug nanocrystals. J. Pharm..

[64] Martena V., Censi R., Hoti E., Malaj L., Di Martino P. (2012). Indomethacin nanocrystals prepared by different laboratory scale methods: Effect on crystalline form and dissolution behavior. J. Nanopart. Res..

[65] Van Eerdenbrugh B., Froyen L., Van Humbeeck J., Martens J.A., Augustijns P., Van den Mooter G. (2008). Drying of crystalline drug nanosuspensions—The importance of surface hydrophobicity on dissolution behavior upon redispersion. Eur. J. Pharm. Sci..

[66] Choi J.-Y., Park C.H., Lee J. (2008). Effect of polymer molecular weight on nanocomminution of poorly soluble drug. Drug Deliv..

[67] Kim S., Lee J. (2010). Effective polymeric dispersants for vacuum, convection and freeze drying of drug nanosuspensions. Int. J. Pharm..

[68] Chan H.-K., Kwok P.C.L. (2011). Production methods for nanodrug particles using the bottom-up approach. Adv. Drug Deliv. Rev..

[69] Gomez A., Bingham D., Juan L.D., Tang K. (1998). Production of protein nanoparticles by electrospray drying. J. Aerosol Sci..

[70] Basa S., Muniyappan T., Karatgi P., Prabhu R., Pillai R. (2008). Production and in vitro characterization of solid dosage form incorporating drug nanoparticles. Drug Dev. Ind. Pharm..

[71] Bhakay A., Davé R., Bilgili E. (2013). Recovery of bcs class II drugs during aqueous redispersion of core–shell type nanocomposite particles produced via fluidized bed coating. Powder Technol..

[72] Krull S.M., Ammirata J., Bawa S., Li M., Bilgili E., Davé R.N. (2017). Critical material attributes of strip films loaded with poorly water-soluble drug nanoparticles: II. Impact of polymer molecular weight. J. Pharm. Sci..

[73] Krull S.M., Moreno J., Li M., Bilgili E., Davé R.N. (2017). Critical material attributes (cmas) of strip films loaded with poorly water-soluble drug nanoparticles: III. Impact of drug nanoparticle loading. Int. J. Pharm..

[74] Krull S.M., Patel H.V., Li M., Bilgili E., Davé R.N. (2016). Critical material attributes (cmas) of strip films loaded with poorly water-soluble drug nanoparticles: I. Impact of plasticizer on film properties and dissolution. Eur. J. Pharm. Sci..

[75] Sievens-Figueroa L., Bhakay A., Jerez-Rozo J.I., Pandya N., Romañach R.J., Michniak-Kohn B., Iqbal Z., Bilgili E., Davé R.N. (2012). Preparation and characterization of hydroxypropyl methyl cellulose films containing stable bcs class ii drug nanoparticles for pharmaceutical applications. Int. J. Pharm..

[76] Susarla R., Afolabi A., Patel D., Bilgili E., Davé R.N. (2015). Novel use of superdisintegrants as viscosity enhancing agents in biocompatible polymer films containing griseofulvin nanoparticles. Powder Technol..

[77] Baumgartner R., Eitzlmayr A., Matsko N., Tetyczka C., Khinast J., Roblegg E. (2014). Nano-extrusion: A promising tool for continuous manufacturing of solid nano-formulations. Int. J. Pharm..

[78] Khinast J., Baumgartner R., Roblegg E. (2013). Nano-extrusion: A one-step process for manufacturing of solid nanoparticle formulations directly from the liquid phase. AAPS PharmSciTech.

[79] Li M., Ioannidis N., Gogos C., Bilgili E. (2017). A comparative assessment of nanocomposites vs. *Amorphous* solid dispersions prepared via nanoextrusion for drug dissolution enhancement. Eur. J. Pharm. Biopharm..

[80] Ye X., Patil H., Feng X., Tiwari R.V., Lu J., Gryczke A., Kolter K., Langley N., Majumdar S., Neupane D. (2016). Conjugation of hot-melt extrusion with high-pressure homogenization: A novel method of continuously preparing nanocrystal solid dispersions. AAPS PharmSciTech.

[81] Zhang H., Hu H., Zhang H., Dai W., Wang X., Wang X., Zhang Q. (2015). Effects of pegylated paclitaxel nanocrystals on breast cancer and its lung metastasis. Nanoscale.

[82] Chen J.-F., Zhang J.-Y., Shen Z.-G., Zhong J., Yun J. (2006). Preparation and characterization of amorphous cefuroxime axetil drug nanoparticles with novel technology: High-gravity antisolvent precipitation. Ind. Eng. Chem. Res..

[83] Zhong J., Shen Z., Yang Y., Chen J. (2005). Preparation and characterization of uniform nanosized cephradine by combination of reactive precipitation and liquid anti-solvent precipitation under high gravity environment. Int. J. Pharm..

[84] Zhao H., Wang J.-X., Wang Q.-A., Chen J.-F., Yun J. (2007). Controlled liquid antisolvent precipitation of hydrophobic pharmaceutical nanoparticles in a microchannel reactor. Ind. Eng. Chem. Res..

[85] Chiou H., Li L., Hu T., Chan H.K., Chen J.F., Yun J. (2007). Production of salbutamol sulfate for inhalation by high-gravity controlled antisolvent precipitation. Int. J. Pharm..

[86] Chiou H., Chan H.K., Heng D., Prud’homme R.K., Raper J.A. (2008). A novel production method for inhalable cyclosporine a powders by confined liquid impinging jet precipitation. J. Aerosol Sci..

[87] Kakran M., Shegokar R., Sahoo N.G., Gohla S., Li L., Muller R.H. (2012). Long-term stability of quercetin nanocrystals prepared by different methods. J. Pharm. Pharmacol..

[88] Kobierski S., Ofori-Kwakye K., Müller R., Keck C. (2009). Resveratrol nanosuspensions for dermal application–production, characterization, and physical stability. Die Pharm. Int. J. Pharm. Sci..

[89] Lemke A., Kiderlen A.F., Petri B., Kayser O. (2010). Delivery of amphotericin b nanosuspensions to the brain and determination of activity against balamuthia mandrillaris amebas. Nanomed. Nanotechnol. Biol. Med..

[90] Mishra P.R., Al Shaal L., Müller R.H., Keck C.M. (2009). Production and characterization of hesperetin nanosuspensions for dermal delivery. Int. J. Pharm..

[91] Sharma P., Denny W.A., Garg S. (2009). Effect of wet milling process on the solid state of indomethacin and simvastatin. Int. J. Pharm..

[92] Al Shaal L., Shegokar R., Müller R.H. (2011). Production and characterization of antioxidant apigenin nanocrystals as a novel uv skin protective formulation. Int. J. Pharm..

[93] Martena V., Censi R., Hoti E., Malaj L., Di Martino P. (2013). Preparation of glibenclamide nanocrystals by a simple laboratory scale ultra cryo-milling. J. Nanopart. Res..

[94] Dressman J., Butler J., Hempenstall J., Reppas C. (2001). The bcs: Where do we go from here?. Pharm. Technol..

[95] Noyes A.A., Whitney W.R. (1897). The rate of solution of solid substances in their own solutions. J. Am. Chem. Soc..

[96] Muller R. (2004). Drug nanocrystals of poorly soluble drugs. Encycl. Nanosci. Nanotechnol..

[97] Gao L., Zhang D., Chen M. (2008). Drug nanocrystals for the formulation of poorly soluble drugs and its application as a potential drug delivery system. J. Nanopart. Res..

[98] Junyaprasert V.B., Morakul B. (2015). Nanocrystals for enhancement of oral bioavailability of poorly water-soluble drugs. Asian J. Pharm. Sci..

[99] Moschwitzer J., Muller R. (2007). Drug nanocrystals-the universal formulation approach for poorly soluble drugs. Drugs Pharm. Sci..

[100] Ponchel G., Montisci M.-J., Dembri A., Durrer C., Duchêne D. (1997). Mucoadhesion of colloidal particulate systems in the gastro-intestinal tract. Eur. J. Pharm. Biopharm..

[101] Jacobs C., Kayser O., Muller R.H. (2001). Production and characterisation of mucoadhesive nanosuspensions for the formulation of bupravaquone. Int. J. Pharm..

[102] Delie F. (1998). Evaluation of nano-and microparticle uptake by the gastrointestinal tract. Adv. Drug Deliv. Rev..

[103] Des Rieux A., Fievez V., Garinot M., Schneider Y.-J., Préat V. (2006). Nanoparticles as potential oral delivery systems of proteins and vaccines: A mechanistic approach. J. Control. Release.

[104] Grama C., Ankola D., Kumar M.R. (2011). Poly(lactide-co-glycolide) nanoparticles for peroral delivery of bioactives. Curr. Opin. Colloid Interface Sci..

[105] Gao L., Liu G., Ma J., Wang X., Zhou L., Li X. (2012). Drug nanocrystals: In vivo performances. J. Control. Release.

[106] Liu F., Park J.Y., Zhang Y., Conwell C., Liu Y., Bathula S.R., Huang L. (2010). Targeted cancer therapy with novel high drug-loading nanocrystals. J. Pharm. Sci..

[107] Wang H., Zhu W., Huang Y., Li Z., Jiang Y., Xie Q. (2017). Facile encapsulation of hydroxycamptothecin nanocrystals into zein-based nanocomplexes for active targeting in drug delivery and cell imaging. Acta Biomater..

[108] Chen M.-L., John M., Lee S.L., Tyner K.M. (2017). Development considerations for nanocrystal drug products. AAPS J..

[109] Fu Q., Sun J., Zhang D., Li M., Wang Y., Ling G., Liu X., Sun Y., Sui X., Luo C. (2013). Nimodipine nanocrystals for oral bioavailability improvement: Preparation, characterization and pharmacokinetic studies. Colloids Surf. B Biointerfaces.

[110] Sarnes A., Kovalainen M., Häkkinen M.R., Laaksonen T., Laru J., Kiesvaara J., Ilkka J., Oksala O., Rönkkö S., Järvinen K. (2014). Nanocrystal-based per-oral itraconazole delivery: Superior in vitro dissolution enhancement versus sporanox^®^ is not realized in in vivo drug absorption. J. Control. Release.

[111] Ghosh I., Schenck D., Bose S., Ruegger C. (2012). Optimization of formulation and process parameters for the production of nanosuspension by wet media milling technique: Effect of vitamin e tpgs and nanocrystal particle size on oral absorption. Eur. J. Pharm. Sci..

[112] Niwa T., Miura S., Danjo K. (2011). Universal wet-milling technique to prepare oral nanosuspension focused on discovery and preclinical animal studies—Development of particle design method. Int. J. Pharm..

[113] Tuomela A., Laaksonen T., Laru J., Antikainen O., Kiesvaara J., Ilkka J., Oksala O., Rönkkö S., Järvinen K., Hirvonen J. (2015). Solid formulations by a nanocrystal approach: Critical process parameters regarding scale-ability of nanocrystals for tableting applications. Int. J. Pharm..

[114] Hollis C.P., Weiss H.L., Evers B.M., Gemeinhart R.A., Li T. (2014). In vivo investigation of hybrid paclitaxel nanocrystals with dual fluorescent probes for cancer theranostics. Pharm. Res..

[115] Müller R.H., Gohla S., Keck C.M. (2011). State of the art of nanocrystals—Special features, production, nanotoxicology aspects and intracellular delivery. Eur. J. Pharm. Biopharm..

[116] Ventola C.L. (2017). Progress in nanomedicine: Approved and investigational nanodrugs. Pharm. Ther..

[117] Havel H.A. (2016). Where are the nanodrugs? An industry perspective on development of drug products containing nanomaterials. AAPS J..

[118] Gaul R., Ramsey J.M., Heise A., Cryan S.-A., Greene C.M. (2018). Nanotechnology approaches to pulmonary drug delivery: Targeted delivery of small molecule and gene-based therapeutics to the lung. Design of Nanostructures for Versatile Therapeutic Applications.

[119] Araújo J., Gonzalez E., Egea M.A., Garcia M.L., Souto E.B. (2009). Nanomedicines for ocular nsaids: Safety on drug delivery. Nanomed. Nanotechnol. Biol. Med..

[120] Wang X., Wang S., Zhang Y. (2016). Advance of the application of nano-controlled release system in ophthalmic drug delivery. Drug Deliv..

[121] Kassem M., Rahman A.A., Ghorab M., Ahmed M., Khalil R. (2007). Nanosuspension as an ophthalmic delivery system for certain glucocorticoid drugs. Int. J. Pharm..

[122] Tuomela A., Liu P., Puranen J., Rönkkö S., Laaksonen T., Kalesnykas G., Oksala O., Ilkka J., Laru J., Järvinen K. (2014). Brinzolamide nanocrystal formulations for ophthalmic delivery: Reduction of elevated intraocular pressure in vivo. Int. J. Pharm..

[123] Petersen R. (2006). Nanocrystals for Use in Topical Formulations and Method of Production Thereof. Germany Patent.

[124] Bahl D., Bogner R.H. (2006). Amorphization of indomethacin by co-grinding with neusilin us2: Amorphization kinetics, physical stability and mechanism. Pharm. Res..

[125] Blagden N., de Matas M., Gavan P.T., York P. (2007). Crystal engineering of active pharmaceutical ingredients to improve solubility and dissolution rates. Adv. Drug Deliv. Rev..

[126] Martino P.D., Magnoni F., Peregrina D.V., Gigliobianco M.R., Censi R., Malaj L. (2016). Formation, physicochemical characterization, and thermodynamic stability of the amorphous state of drugs and excipients. Curr. Pharm. Des..

[127] Niwa T., Nakanishi Y., Danjo K. (2010). One-step preparation of pharmaceutical nanocrystals using ultra cryo-milling technique in liquid nitrogen. Eur. J. Pharm. Sci..

[128] Willart J.F., Descamps M. (2008). Solid state amorphization of pharmaceuticals. Mol. Pharm..

[129] Zhang G.G.Z., Law D., Schmitt E.A., Qiu Y. (2004). Phase transformation considerations during process development and manufacture of solid oral dosage forms. Adv. Drug Deliv. Rev..

[130] Derjaguin B., Landau L. (1993). Theory of the stability of strongly charged lyophobic sols and of the adhesion of strongly charged particles in solutions of electrolytes. Prog. Surf. Sci..

[131] Verwey E.J.W., Overbeek J.T.G. (1946). Long distance forces acting between colloidal particles. Trans. Faraday Soc..

[132] Van Eerdenbrugh B., Van den Mooter G., Augustijns P. (2008). Top-down production of drug nanocrystals: Nanosuspension stabilization, miniaturization and transformation into solid products. Int. J. Pharm..

[133] Peltonen L., Hirvonen J. (2010). Pharmaceutical nanocrystals by nanomilling: Critical process parameters, particle fracturing and stabilization methods. J. Pharm. Pharmacol..

[134] Mu S., Li M., Guo M., Yang W., Wang Y., Li J., Fu Q., He Z. (2016). Spironolactone nanocrystals for oral administration: Different pharmacokinetic performances induced by stabilizers. Colloids Surf. B Biointerfaces.

[135] Choi J.-Y., Yoo J.Y., Kwak H.-S., Uk Nam B., Lee J. (2005). Role of polymeric stabilizers for drug nanocrystal dispersions. Curr. Appl. Phys..

[136] Liu P., Rong X., Laru J., van Veen B., Kiesvaara J., Hirvonen J., Laaksonen T., Peltonen L. (2011). Nanosuspensions of poorly soluble drugs: Preparation and development by wet milling. Int. J. Pharm..

[137] Hancock B.C., Zografi G. (1997). Characteristics and significance of the amorphous state in pharmaceutical systems. J. Pharm. Sci..

[138] Brough C., Williams R.O. (2013). Amorphous solid dispersions and nano-crystal technologies for poorly water-soluble drug delivery. Int. J. Pharm..

[139] Liversidge G.G., Cundy K.C., Bishop J.F., Czekai D.A., STWB Inc. (1991). Czekai Surface Modified Drug Nanoparticles. U.S. Patent.

[140] Kayaert P., Van den Mooter G. (2012). Is the amorphous fraction of a dried nanosuspension caused by milling or by drying? A case study with naproxen and cinnarizine. Eur. J. Pharm. Biopharm..

[141] Shakhtshneider T.P., Danède F., Capet F., Willart J.F., Descamps M., Paccou L., Surov E.V., Boldyreva E.V., Boldyrev V.V. (2007). Grinding of drugs with pharmaceutical excipients at cryogenic temperatures. J. Therm. Anal. Calorim..

[142] Xiong R., Lu W., Li J., Wang P., Xu R., Chen T. (2008). Preparation and characterization of intravenously injectable nimodipine nanosuspension. Int. J. Pharm..

[143] Lee J., Lee S.-J., Choi J.-Y., Yoo J.Y., Ahn C.-H. (2005). Amphiphilic amino acid copolymers as stabilizers for the preparation of nanocrystal dispersion. Eur. J. Pharm. Sci..

[144] Lu Y., Wang Z.-H., Li T., McNally H., Park K., Sturek M. (2014). Development and evaluation of transferrin-stabilized paclitaxel nanocrystal formulation. J. Control. Release.

[145] Thanki K., Gangwal R.P., Sangamwar A.T., Jain S. (2013). Oral delivery of anticancer drugs: Challenges and opportunities. J. Control. Release.

[146] Müller R.H., Jacobs C., Kayser O. (2001). Nanosuspensions as particulate drug formulations in therapy: Rationale for development and what we can expect for the future. Adv. Drug Deliv. Rev..

[147] Gao L., Zhang D., Chen M., Duan C., Dai W., Jia L., Zhao W. (2008). Studies on pharmacokinetics and tissue distribution of oridonin nanosuspensions. Int. J. Pharm..

[148] Fuhrmann K., Schulz J.D., Gauthier M.A., Leroux J.-C. (2012). Peg nanocages as non-sheddable stabilizers for drug nanocrystals. ACS Nano.

[149] Shubar H.M., Lachenmaier S., Heimesaat M.M., Lohman U., Mauludin R., Mueller R.H., Fitzner R., Borner K., Liesenfeld O. (2011). Sds-coated atovaquone nanosuspensions show improved therapeutic efficacy against experimental acquired and reactivated toxoplasmosis by improving passage of gastrointestinal and blood-brain barriers. J. Drug Target..

[150] Pandey G., Mittapelly N., Banala V.T., Mishra P.R. (2018). Multifunctional glycoconjugate assisted nanocrystalline drug delivery for tumor targeting and permeabilization of lysosomal-mitochondrial membrane. ACS Appl. Mater. Interfaces.

[151] Rao Y.M., Kumar M.P., Apte S. (2008). Formulation of nanosuspensions of albendazole for oral administration. Curr. Nanosci..

[152] Langguth P., Hanafy A., Frenzel D., Grenier P., Nhamias A., Ohlig T., Vergnault G., Spahn-Langguth H. (2005). Nanosuspension formulations for low-soluble drugs: Pharmacokinetic evaluation using spironolactone as model compound. Drug Dev. Ind. Pharm..

[153] Rachmawati H., Al Shaal L., Müller R.H., Keck C.M. (2013). Development of curcumin nanocrystal: Physical aspects. J. Pharm. Sci..

[154] Ravichandran R. (2012). Development of an oral curcumin nanocrystal formulation. J. Nanotechnol. Eng. Med..

[155] Moorthi C., Kathiresan K. (2013). Fabrication of highly stable sonication assisted curcumin nanocrystals by nanoprecipitation method. Drug Invent. Today.

[156] Quan P., Shi K., Piao H., Piao H., Liang N., Xia D., Cui F. (2012). A novel surface modified nitrendipine nanocrystals with enhancement of bioavailability and stability. Int. J. Pharm..

[157] Koradia K.D., Parikh R.H., Koradia H.D. (2018). Albendazole nanocrystals: Optimization, spectroscopic, thermal and anthelmintic studies. J. Drug Deliv. Sci. Technol..

[158] Xia D., Quan P., Piao H., Piao H., Sun S., Yin Y., Cui F. (2010). Preparation of stable nitrendipine nanosuspensions using the precipitation-ultrasonication method for enhancement of dissolution and oral bioavailability. Eur. J. Pharm. Sci..

[159] Quan P., Xia D., Piao H., Piao H., Shi K., Jia Y., Cui F. (2011). Nitrendipine nanocrystals: Its preparation, characterization, and in vitro—In vivo evaluation. AAPS PharmSciTech.

[160] Kakran M., Shegokar R., Sahoo N.G., Al Shaal L., Li L., Müller R.H. (2012). Fabrication of quercetin nanocrystals: Comparison of different methods. Eur. J. Pharm. Biopharm..

[161] Kobierski S., Ofori-Kwakye K., Müller R., Keck C. (2011). Resveratrol nanosuspensions: Interaction of preservatives with nanocrystal production. Die Pharm. Int. J. Pharm. Sci..

[162] Bi Y., Liu J., Wang J., Hao J., Li F., Wang T., Sun H.W., Guo F. (2015). Particle size control and the interactions between drug and stabilizers in an amorphous nanosuspension system. J. Drug Deliv. Sci. Technol..

[163] Onoue S., Takahashi H., Kawabata Y., Seto Y., Hatanaka J., Timmermann B., Yamada S. (2010). Formulation design and photochemical studies on nanocrystal solid dispersion of curcumin with improved oral bioavailability. J. Pharm. Sci..

[164] Döge N., Hönzke S., Schumacher F., Balzus B., Colombo M., Hadam S., Rancan F., Blume-Peytavi U., Schäfer-Korting M., Schindler A. (2016). Ethyl cellulose nanocarriers and nanocrystals differentially deliver dexamethasone into intact, tape-stripped or sodium lauryl sulfate-exposed ex vivo human skin-assessment by intradermal microdialysis and extraction from the different skin layers. J. Control. Release.

[165] Pireddu R., Caddeo C., Valenti D., Marongiu F., Scano A., Ennas G., Lai F., Fadda A.M., Sinico C. (2016). Diclofenac acid nanocrystals as an effective strategy to reduce in vivo skin inflammation by improving dermal drug bioavailability. Colloids Surf. B Biointerfaces.

[166] Pireddu R., Sinico C., Ennas G., Marongiu F., Muzzalupo R., Lai F., Fadda A.M. (2015). Novel nanosized formulations of two diclofenac acid polymorphs to improve topical bioavailability. Eur. J. Pharm. Sci..

[167] Dhapte V., Pokharkar V. (2011). Polyelectrolyte stabilized antimalarial nanosuspension using factorial design approach. J. Biomed. Nanotechnol..

[168] Hecq J., Deleers M., Fanara D., Vranckx H., Amighi K. (2005). Preparation and characterization of nanocrystals for solubility and dissolution rate enhancement of nifedipine. Int. J. Pharm..

[169] Hecq J., Nollevaux G., Deleers M., Fanara D., Vranckx H., Peulen O., Dandrifosse G., Amighi K. (2006). Nifedipine nanocrystals: Pharmacokinetic evaluation in the rat and permeability studies in caco-2/ht29-5 m21 (co)-cultures. J. Drug Deliv. Sci. Technol..

[170] Yang H., Teng F., Wang P., Tian B., Lin X., Hu X., Zhang L., Zhang K., Zhang Y., Tang X. (2014). Investigation of a nanosuspension stabilized by soluplus(r) to improve bioavailability. Int. J. Pharm..

[171] George M., Ghosh I. (2013). Identifying the correlation between drug/stabilizer properties and critical quality attributes (cqas) of nanosuspension formulation prepared by wet media milling technology. Eur. J. Pharm. Sci..

[172] Varshosaz J., Ahmadipour S., Tabbakhian M., Ahmadipour S. (2018). Nanocrystalization of pioglitazone by precipitation method. Drug Res..

[173] Lai F., Pini E., Angioni G., Manca M.L., Perricci J., Sinico C., Fadda A. (2011). Nanocrystals as tool to improve piroxicam dissolution rate in novel orally disintegrating tablets. Eur. J. Pharm. Biopharm..

[174] Iurian S., Bogdan C., Tomuță I., Szabó-Révész P., Chvatal A., Leucuța S.E., Moldovan M., Ambrus R. (2017). Development of oral lyophilisates containing meloxicam nanocrystals using qbd approach. Eur. J. Pharm. Sci..

[175] Tan E.H., Parmentier J., Low A., Möschwitzer J.P. (2017). Downstream drug product processing of itraconazole nanosuspension: Factors influencing tablet material properties and dissolution of compacted nanosuspension-layered sugar beads. Int. J. Pharm..

[176] Shariare M.H., Sharmin S., Jahan I., Reza H., Mohsin K. (2018). The impact of process parameters on carrier free paracetamol nanosuspension prepared using different stabilizers by antisolvent precipitation method. J. Drug Deliv. Sci. Technol..

[177] Chen C., Wang L., Cao F., Miao X., Chen T., Chang Q., Zheng Y. (2016). Formulation of 20 (s)-protopanaxadiol nanocrystals to improve oral bioavailability and brain delivery. Int. J. Pharm..

[178] Yi T., Tang D., Wang F., Zhang J., Zhang J., Wang J., Xu X., Zhang J. (2017). Enhancing both oral bioavailability and brain penetration of puerarin using borneol in combination with preparation technologies. Drug Deliv..

[179] Liu Y., Ma Y., Xu J., Chen Y., Xie J., Yue P., Zheng Q., Yang M. (2017). Apolipoproteins adsorption and brain-targeting evaluation of baicalin nanocrystals modified by combination of tween80 and tpgs. Colloids Surf. B Biointerfaces.

[180] Karmali P.P., Kotamraju V.R., Kastantin M., Black M., Missirlis D., Tirrell M., Ruoslahti E. (2009). Targeting of albumin-embedded paclitaxel nanoparticles to tumors. Nanomed. Nanotechnol. Biol. Med..

[181] Arias J.L., Reddy L.H., Couvreur P. (2008). Magnetoresponsive squalenoyl gemcitabine composite nanoparticles for cancer active targeting. Langmuir.

[182] Kim S., Lee J. (2011). Folate-targeted drug-delivery systems prepared by nano-comminution. Drug Dev. Ind. Pharm..

[183] Zhan H., Liang J.F. (2016). Extreme activity of drug nanocrystals coated with a layer of non-covalent polymers from self-assembled boric acid. Sci. Rep..

